# Scabies outbreak management in a vulnerable elderly population: Evidence from northeastern Iran

**DOI:** 10.1016/j.parepi.2026.e00524

**Published:** 2026-07-03

**Authors:** Hamed Behniafar, Kiana Yahyaei, Arian Taghdiri, Hamideh Mohammadzadeh, Hossein Pazoki

**Affiliations:** a Department of Medical Parasitology, Sarab Faculty of Medical Sciences, Sarab, Iran; b Student Research Committee, Gonabad University of Medical Sciences, Gonabad, Iran; c Department of Dermatology, Gonabad University of Medical Sciences, Gonabad, Iran; d Faculty of Medicine, Department of Medical Microbiology, Infectious Diseases Research Center, Gonabad University of Medical Science, Gonabad, Iran

**Keywords:** *Sarcoptes scabiei*, Outbreak control, Nursing home, Iran

## Abstract

Infestation with *Sarcoptes scabiei* is a highly contagious, neglected tropical disease affecting vulnerable individuals in institutional settings. The present study described an outbreak at a 24-h elderly care facility in northeastern Iran with emphasis on clinical characteristics, management, and long-term consequences. From December 22, 2023, to February 21, 2024, 24 elderly participants and 13 participating staff members were screened after an index case was diagnosed with Norwegian scabies. The attack rate among residents was 16.7% (4/24; 95% CI 6.7–35.9%), while no staff members were laboratory-confirmed positive (0/13; 95% CI 0–22.8%). The latter diagnosis was performed for the elderly and staff based on the 2020 International Alliance for the Control of Scabies (IACS) criteria and confirmed by microscopic examination. Treatment included 5% permethrin cream and oral ivermectin, both repeated at one-week intervals, whereas unaffected individuals received permethrin prophylaxis. Environmental control strategies, including hot laundering, vacuuming, and isolation, were employed. Four cases were identified, including one of crusted scabies and three of ordinary scabies; all affected individuals were female. Complete recovery occurred within 8 to 19 days, with no new cases reported during the one-year follow-up. The outbreak was effectively managed by taking advantage of early diagnosis, combined therapy, prevention, and environmental measures. The current study highlighted the necessity of complex protocols in nursing homes and similar centers to control scabies in low-endemic areas.

## 1 Introduction

*Sarcoptes scabiei* var. *hominis* is an ectoparasitic mite, and its infestation can cause two highly communicable infectious dermatological conditions that are named ordinary scabies (common scabies, classical scabies, OS) and crusted scabies (formerly Norwegian scabies, CS). The distinction between these two conditions lies in the variation of clinical symptoms and the quantity of mites infesting the patient (Matthews et al., 2021). Vast hyperkeratotic lesions and a heavy burden of the mites characterize CS, and this form is more severe and contagious than OS (Al-Soufi et al., 2025). Specifically, to be more precise, CS causes more severe symptoms than OS, including thick hyperkeratosis, extensive scaling, and crusty lesions that can be loose and flaky or thick and sticky (Agyei et al., 2024; Niode et al., 2022).

Infection with this ectoparasite is among the most common and high-morbidity skin diseases, especially in developing countries (Sunderkötter et al., 2016). According to data from the World Health Organization (Park et al., 2019) According to the official website, over 200 million individuals are affected by *Sarcoptes* at any given time, with more than 400 million new cases reported annually worldwide (Organization, W.H, 2023). Consequently, the WHO has designated scabies as a neglected tropical disease since 2017 (Engelman et al., 2021)On the other hand, outbreaks of OS and CS primarily affect health institutions and vulnerable communities in developed countries (Thomas et al., 2017).

Scabies remains a public health issue in Iran, with a pooled prevalence of approximately 7% (95% CI 4.7–10.3%) between 2000 and 2022. Prevalence varies by region, population, and socioeconomic factors, with higher rates in schoolchildren and institutional settings. Recent studies highlight ongoing transmission due to overcrowding, limited healthcare access, and seasonal patterns, necessitating targeted outbreak management in vulnerable groups like the elderly in care facilities (Khoobdel et al., 2022; Sanei-Dehkordi et al., 2021).

The mite is classified into the phylum Arthropoda, class Arachnida, order Acari, and superfamily Sarcoptidae (Niode et al., 2022). In the life cycle of human scabies, gravid female mites burrow into and lay their eggs there, and the larvae mature within two weeks and continue the life cycle. Transmission occurs primarily through direct skin-to-skin contact, but indirect transmission may also occur, as mites can survive on contaminated surfaces for up to 36 h, depending on environmental conditions such as temperature and humidity. However, previous studies suggest indirect transmission is not common except in severe CS cases, where high parasite loads may enhance the risk of transmission by indirect mechanisms, including soiled bedding or clothing (Walton and Currie, 2007).

Recent revisions in international diagnostic criteria and the designation of scabies as a neglected tropical disease by the World Health Organization have underscored the urgent need for standardized diagnostic approaches and coordinated control strategies(Engelman et al., 2021). Long-term care facilities and other closed settings have been repeatedly identified as hotspots for outbreaks, where delayed recognition and the absence of harmonized protocols can facilitate the rapid transmission of infections among vulnerable populations(White et al., 2016; Lee et al., 2020). Taken together, these developments highlight the ongoing challenges of scabies management and the need for context-specific evidence. The present study contributes to this field by documenting an outbreak in an elderly care facility and illustrating practical measures that proved effective in its control.

## 2 Materials and methods

### 2.1 Study area

The present study was conducted in a 24-h care center for the elderly in northeastern Iran between December 22, 2023, and February 21, 2024. In this center, 13 staff members, including administrative staff, doctors, and nurses, care for 24 older adults.

### 2.2 Index case

**Fig. 1:**
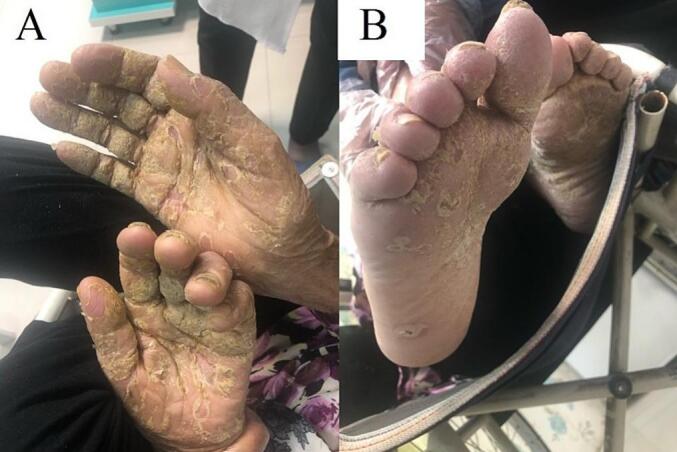
Clinical features of crusted (Norwegian) scabies in an elderly patient from a nursing home outbreak in Northeastern Iran. A) extensive crusted lesions and scaling on the hands and fingers, B) hyperkeratotic lesions with scaling on the feet.

**Fig. 2:**
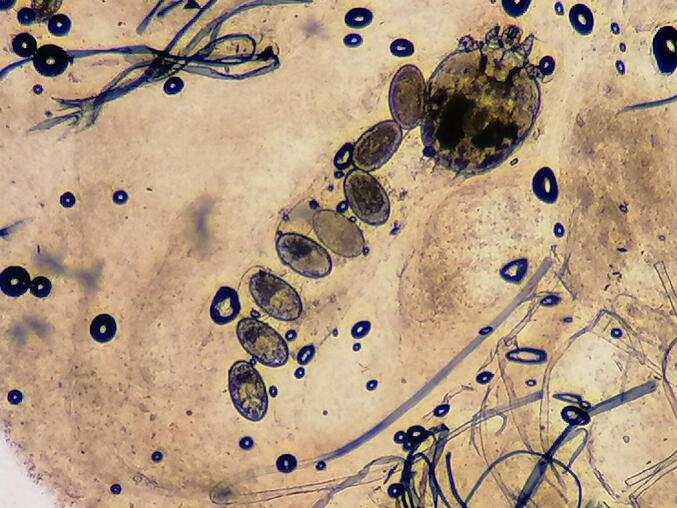
Light microscopic view of an adult female *Sarcoptes scabiei* with eggs (10% KOH wet mount, Olympus CX23, ×400). The specimen was obtained from an elderly patient in a care facility in Northeastern Iran.

The index case of scabies at this center (Case A) was an 84-year-old woman. On December 22, 2023 (Day 0), the Gonabad University of Medical Sciences Office of Health was informed of 1 case (Case A) with skin symptoms in an elderly nursing home resident referred to the laboratory (Fig. 1). The preliminary diagnosis was based on suspected symptoms of scabies and was confirmed by microscopic examination (Fig. 2).

### 2.3 Management of the outbreak

**Fig. 3:**
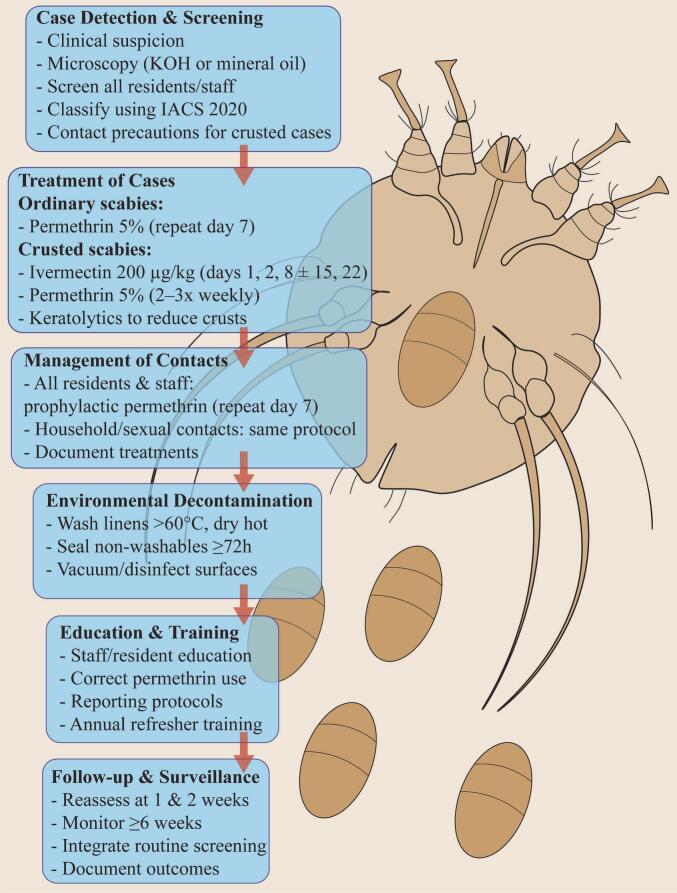
Flowchart detailing the systematic protocol for managing the scabies outbreak in the nursing home.

In response to the scabies outbreak, a comprehensive management protocol was established, which included initial case detection, case grading, mass treatment for all residents and staff, outbreak control measures, and ongoing follow-up and surveillance (Fig. 3).

#### 2.3.1 Case and outbreak detection

After the cases were reported to the Public Health Department, the signs and symptoms of scabies infection were explained to all residents and staff. Residents of the nursing home and staff caring for them were advised to report any symptoms that might indicate a scabies infection for evaluation by the nursing home's general practitioner. Staff were also advised to see their general practitioner if they developed any symptoms.

Additionally, immediate investigations were conducted at the center. Therefore, the rest of the elderly residents and staff were examined, and three more cases were diagnosed. All residents and staff underwent initial clinical screening for pruritus, burrows, papules, or crusted lesions. During the screening process, four residents presented with pruritus and compatible skin lesions suggestive of scabies. No staff members reported persistent pruritus or showed compatible lesions during clinical evaluation. Skin scrapings were performed on symptomatic individuals or close contacts from active lesion margins (interdigital spaces, wrists, flexural areas) using a sterile scalpel after 70% alcohol disinfection. Specimens were examined in 10% KOH under light microscopy. Diagnosis followed the in the 2020 International Alliance for the Control of Scabies (IACS) consensus criteria, which classify scabies into three levels of diagnostic certainty: Confirmed (Level A), Clinical (Level B, indicating probable cases), and Suspected (Level C, indicating possible cases) (Engelman et al., 2020). In this outbreak, all four cases meeting clinical criteria were microscopically confirmed positive; No clinically suspected symptomatic cases yielded negative microscopic results during the investigation. Dermoscopy was not used in this study because the equipment was not available at the facility during the outbreak investigation. Although dermoscopy is a less invasive and valuable screening tool, skin scraping with microscopic examination was chosen as it provides definitive confirmation of *Sarcoptes scabiei* (Level A diagnosis per IACS criteria) with high specificity. This was particularly important in an institutional outbreak setting to guide targeted treatment, isolation, and prophylaxis decisions. Scrapings were limited to symptomatic or high-risk individuals to minimize patient discomfort.

The outbreak of scabies was confirmed based on the identified cases, and health and medical entomology experts were dispatched to the site to control it. Efforts included thorough inspections, public education on prevention measures, and the implementation of treatment protocols to mitigate the spread of scabies within the affected population.

After confirming case A through additional testing (microscopic confirmation), all elderly residents and staff who had close contact with them were sampled and examined. Skin scrapings were taken from the active margins of burrows or erythematous papules, most often from the interdigital spaces, wrists, or flexural areas. After disinfecting the skin with 70% alcohol, the edges of the selected lesions were gently scraped with a sterile disposable scalpel. Each specimen was placed on a glass slide and mixed with a drop of 10% potassium hydroxide (Takeda et al., 2017), which was used to clear keratin and enhance visualization of mites, eggs, and scybala without staining (Meletis et al., 2018). The preparations were covered with a coverslip and examined promptly (within 15 min) to prevent drying artifacts. For each patient, three slides were prepared and examined under a light microscope (Olympus CX23) at ×100 and ×400 magnification. The study parasitologist reviewed all slides.

#### 2.3.2 Case grading

The degree of scabies severity was determined by the number of skin lesions and the clinical pattern of the skin lesions. Grade 1 was defined as mild scabies with few (typical) lesions, minimal crusting, few burrows, and mild itching. Grade 2 scabies was moderate with more extensive lesions, heavier burrowing, moderate crusting, and significant pruritus. Grade 3 corresponded to the most severe form, i.e., crusted scabies, characterized by extensive, thick, hyperkeratotic crusting over large parts of the body, as well as deep fissuring of the skin, bleeding, and extensive purulent exudate resulting from secondary bacterial infections. This serious category correlated with high mite load, damage to the skin, and sometimes repeated previous episodes/hospital admissions. In this grading system, treatment was based on the severity of the disease, which was assessed, with the grades increasing proportionate to the mite load and extent of skin lesion. In microscopic examination, each of the three prepared slides showed numerous adult mites and eggs, with an estimated count of around 10 identifiable organisms per slide. Given that ordinary scabies typically harbors only 10–15 mites on the entire body, whereas crusted scabies can involve thousands to millions of mites, these findings, together with the patient's hyperkeratotic crusted lesions, are consistent with a high mite burden and support the diagnosis of crusted scabies (Engelman et al., 2020).In case of crusted scabies, the severity was additionally subcategorized by degree of crust distribution, crust depth, skin fissuring, and patient history in mild (score 1), moderate (score 2), and severe (score 3) cases (Davis et al., 2013).

#### 2.3.3 Treatment

Immediately after the examinations and diagnosis of the patients, the treatment was carried out as follows. Positive cases were treated with 5% permethrin cream and oral ivermectin tablets at a dosage of 200 μg/kg. Both medications were administered twice, with a one-week interval between doses. Additionally, healthy residents of the nursing home and all employees received 5% permethrin cream for chemoprophylaxis purposes.

#### 2.3.4 Control of the outbreak

To effectively manage the outbreak and mitigate the risk of scabies transmission, it is essential to implement timely and appropriate treatment for affected individuals and eliminate mites from the living environments. Therefore, all clothes, bedding, and towels were laundered in hot water (> 60 °C) and dried at a high temperature; non-washable items were kept in airtight plastic bags for at least 72 h; and the carpets, furniture, and other surfaces were vacuumed carefully, and the bag was disposed of properly. It is worth mentioning that patients are considered recovered when clinical criteria show no new burrows, papules, or lesions, with pruritus substantially reduced or resolved, though residual itching from hypersensitivity may persist for 2–4 weeks post-treatment (Uzun et al., 2024). Microscopic criteria require negative skin scrapings, confirming the absence of mites, eggs, or scybala. In crusted cases common among the elderly, repeat evaluations at 14 and 28 days ensure complete eradication (Uzun et al., 2024).

#### 2.3.5 Follow-up and surveillance

After the initial course of therapy, all patients were re-examined at one and two weeks to assess treatment efficacy and identify any persistent or delayed symptoms. Clinical monitoring was then continued for at least six weeks, corresponding to two complete life cycles of the mite, to detect possible re-infestation or treatment failure. Beyond this period, the facility implemented routine dermatologic examinations for all residents on an annual basis, and nursing staff was instructed to report any suspected new lesions promptly. Medical records and direct communication with caregivers were reviewed throughout the 12-month observation period, during which no additional cases of scabies were identified.

## 3 Results

A total of 24 elderly people, ranging in age from 55 to 94 years and with a mean age of 80.38 years (SD ± 10.2), were receiving service from the nursing home. Most residents were female (*n* = 15, 62.5%) and single (*n* = 20, 83.3%), reflecting the prevalent demographics of elderly residents in long-term care facilities in the study region. In addition, all of them were illiterate, occupationally disabled, and had no history of contact with animals (Table 1).

**Table 1:** Demographic characteristics of nursing home residents and scabies patients.

Parameter	Overall Residents (*n* = 24)	Scabies Patients (*n* = 4)
Age Range (years)	55–94	72–84
Mean Age (years)	80.38 ± 10.2 (SD)	78.5 ± 5.5 (SD)
Gender (Male/Female)	8 (33.3%) / 16 (66.7%)	0/4 (100% female)
Marital Status (Married/Single)	3 (12.5%) / 21 (87.5%)	2/2
Staff Screening Results	Not Applicable	Negative (0/13; 0%, 95% CI: 0.0–22.8%)
One-Year Follow-Up	Not Applicable	No New Cases
Attack Rate (Residents)	4/24 (16.7%; 95% CI: 6.7–35.9%)	–

The reported case and three actively identified cases corresponded to an attack rate of 16.7% among residents (4/24; 95% CI: 6.7–35.9%), while no cases were detected among staff (0/13; 95% CI: 0–22.8%). Of the four cases, one (Case A) presented with Norwegian scabies, and the remaining three had the ordinary type (Table 2). All affected residents were female, with a mean age of 78.5 years (SD ± 5.3); two cases were married, and two were single. None of the 13 staff members at the center was found to have scabies, and follow-up assessments confirmed that no additional cases occurred during the subsequent year.

**Table 2:** Clinical and demographic characteristics of scabies cases in the nursing home.

Case	Type of Scabies	Sites of Signs	Signs Grade	Marital Status	Gender	Age (years)
A	Norwegian scabies	Generalized	3	Single	Female	84
B	Ordinary scabies	Hands	1	Married	Female	76
C	Ordinary scabies	Legs and trunk	2	Single	Female	82
D	Ordinary scabies	Legs, hands, and trunk	2	Married	Female	72

It is worth noting that case A, who had Norwegian scabies, was hospitalized, and the others were isolated and treated in the nursing home under the supervision of a physician. The person with Norwegian scabies recovered after 19 days and was discharged from the hospital to the nursing home. The other three cases recovered completely after eight days, and all their symptoms disappeared.

## 4 Discussion

In the present study, an outbreak of scabies was identified and effectively controlled at a 24-h elderly care center in northeastern Iran from December 22, 2023, to February 21, 2024. During this outbreak, four cases were diagnosed, including one CS (index case) and three OS cases. The index case presented with generalized hyperkeratotic lesions and a high mite load, whereas the remaining cases exhibited local symptoms in one or more areas, including the hands, feet, and trunk. Early diagnosis via clinical and microscopic examinations, followed by treatment with 5% permethrin cream and oral ivermectin, led to the full recovery of all four patients. Additionally, the implementation of effective control methods prevented the spread of the infestation to other residents and staff at the center, leading to successful outbreak management.

The skin scraping method, utilized in this study for diagnosing scabies via microscopic examination of mites, eggs, or fecal pellets, demonstrates variable sensitivity ranging from 56% to 75%, with near-perfect specificity when positive (Debash et al., 2024; Shoukat et al., 2023). Effectiveness hinges on sampling sites, favoring burrows in interdigital webs, wrists, or elbows over papules, which often yield false negatives due to absent mites (Mitchell et al., 2024). Timing is crucial; early infestations (3–6 weeks) exhibit low mite counts, whereas chronic cases involve skin alterations that reduce detectability. Limitations include invasiveness, causing discomfort in elderly patients, operator expertise requirements, and potential sampling errors from mite mobility (Mitchell et al., 2024; Tavoletti et al., 2025).

In outbreaks of scabies in institutions such as nursing homes, seasonality plays a significant role in the transmission of scabies. The winter prevalence of this epidemic in Northeastern Iran may be due to increased crowded indoor gatherings and contact of its residents, which created a suitable environment for *S. scabiei* transmission (Rasti et al., 2017). Epidemiological studies repeatedly have demonstrated the peaks of scabies in colder periods, which is powered by prolonged mite viability at lower temperatures and increased human-to-human contact in confined places (Liu et al., 2016). The geographic location of the outbreak in Northeastern Iran, adjacent to Afghanistan, also underscores the role of regional epidemiology on local disease dynamics (Mazumder et al., 2022). In Afghanistan, scabies is characterized by high prevalence due to overcrowding, poor access to medical care, and socioeconomic unrest (Saraj et al., 2023).

The observation that all four scabies cases in our nursing home outbreak were female, despite a mixed-sex resident population of 24 individuals, is noteworthy. This female predominance aligns with several studies on scabies in nursing home residents. For instance, a UK-based study of ten care homes found that 72% of identified cases were female, although this was not statistically significant when adjusted for the higher proportion of female residents (76%) (Cassell et al., 2018). Additionally, a Japanese claims-based analysis of scabies reported that 66.5% of cases were female, with an average patient age of 77.4 years (Yamaguchi et al., 2024). Some meta-analyses indicate a slight male predominance in global scabies rates (OR 1.19 for males); however, institutional settings such as nursing homes may exhibit an opposite trend due to demographic factors (Gupta et al., 2024). Nursing homes typically have a higher number of female residents, reflecting the increased longevity of women and a higher prevalence of scabies among elderly females in community-based studies (Raffi et al., 2019). Furthermore, behavioral factors may also play a role: female residents may engage in more close contact (such as caregiving) in crowded environments, thereby increasing the risk of transmission (Organization, W.H, 2023).

Equally noteworthy was the absence of infection in the spouses of the two married cases, considering scabies' high transmissibility through prolonged skin-to-skin contact, particularly within families and among sexual partners (Thomas et al., 2020). This lack of transmission aligns with sporadic observations in outbreak guidance, where some close contacts exhibit no symptoms (Organization, W.H, 2023). It is plausible that reduced physical contact among the elderly couples in an institutional setting limited the duration of exposure to levels insufficient for mite transfer, typically around 15–20 min. Alternatively, the partners may have experienced subclinical infestations that were not detected during clinical examinations, or they may have exhibited greater resistance due to variations in immune response or skin barrier integrity (Bergamin et al., 2024). Diagnostic challenges, such as atypical presentations in older adults, may also contribute to this phenomenon (Braun et al., 2020). These findings underscore the complex transmission dynamics within nursing home environments and highlight the imperative for rigorous contact tracing and prophylaxis to prevent the oversight of potential reservoirs.

As previously mentioned, the combination of topical permethrin and oral ivermectin was selected for treating all positive cases of the outbreak. This combination provides superior efficacy compared to monotherapy, particularly for crusted scabies, which is common in the elderly and immunocompromised individuals, minimizing treatment failure and enhancing outbreak control in institutional settings (Absil et al., 2022; Agency, 2025). This approach aligns with recent evidence showing improved clearance rates and cost-effectiveness in vulnerable populations.

Importantly, during this outbreak, permethrin was prescribed to all unaffected residents and staff for prophylaxis. Additionally, environmental controls such as laundering textiles at temperatures exceeding 60 °C and regular surface vacuuming were implemented, effectively halting transmission (Engelman et al., 2021; Agency, 2025). The absence of staff transmission suggests that protective protocols and prophylaxis effectively disrupt transmission chains, offering frameworks for global nursing home policies in the context of increasing post-2020 incidences, where institutional outbreaks contribute significantly to disability-adjusted life years (DALYs) (Li et al., 2024).

Our results align with those from similar institutional outbreaks while providing context-specific insights (Cassell et al., 2018; Hewitt et al., 2015; Morrison et al., 2019). A multicenter prospective analysis of scabies outbreaks across ten UK elderly care homes revealed diagnostic delays and the need for coordinated treatments to address episodes without extensive staff involvement, reflecting our experience of zero staff cases despite proximity (Cassell et al., 2018). Similarly, a 2025 report on a significant nosocomial scabies outbreak in a Korean tertiary hospital, originating from a crusted index case, was managed through mass prophylaxis and decontamination, resulting in low attack rates among healthcare workers due to prompt interventions, consistent with our prophylactic success (Jung et al., 2025). However, differences in outbreak dynamics and trends have been observed; recent studies from Italy document a resurgence of scabies in the post-pandemic period, with cases increasing from 42 in 2020 to 748 in 2024 in Campania, and a similar rise in Lazio following pandemic-related declines (Giorgio et al., 2025; Spaziante et al., 2025). These trends have been attributed to disrupted monitoring and potential permethrin resistance factors not present in our Iranian cohort, where dual therapy ensured resolution (Giorgio et al., 2025). This research contributes to the literature by providing data from a Middle Eastern developing context, where reports of institutional scabies are limited, and validates the 2020 IACS consensus diagnostic criteria in such settings, emphasizing the importance of clinical and microscopic confirmation for accuracy (Engelman et al., 2020; Walker et al., 2020). In contrast to Taiwanese epidemiological data indicating scabies prevalence rates of 3.3–7.3% in southern nursing homes based on 2002 surveys, our outbreak exhibited a 16.7% resident attack rate, highlighting the increased vulnerability of enclosed elderly populations, potentially exacerbated by shared living conditions and comorbidities (Derek Richard et al., 2002; Smith et al., 2002). Additional prevalence estimates from Pacific and Southeast Asian countries further underscore the necessity for tailored strategies in diverse settings (Tsoi et al., 2021).

From a practical perspective, these outcomes address gaps in scabies control by demonstrating how early scratch testing with 10% potassium hydroxide, by updated diagnostic guidelines, can mitigate spread among elderly populations susceptible to atypical presentations due to cognitive or mobility impairments (1,3). By achieving containment without relapse, the study underscores the importance of ongoing surveillance and training in at-risk communities, which could help alleviate the over 200 million prevalent cases worldwide (Engelman et al., 2021; El-Moamly, 2021).

Limitations of the study include its single-center design and relatively modest sample size (24 residents), which may restrict the broader applicability of the findings to larger or more diverse facilities (Hewitt et al., 2015). The reliance on microscopy for diagnosis, without the inclusion of PCR for mite strain typing, may have resulted in the oversight of subtle cases or resistance markers; however, it is noteworthy that no treatment failures were reported (Mayer et al., 2022; Veraldi et al., 2023; Andriantsoanirina et al., 2014). While the follow-up period provided reassuring results, it lacked extended symptom tracking, which may have limited the depth of the efficacy evaluations. Such challenges are common in resource-limited outbreak studies and do not diminish the significance of the observed containment results (Morrison et al., 2019).

Future investigations should focus on multicenter cohorts incorporating genomic sequencing to monitor strains and resistance, in light of the 2022–2025 European reports on challenges related to permethrin (Mayer et al., 2022; Veraldi et al., 2023; Andriantsoanirina et al., 2014). Additionally, exploring sociodemographic predictors within Iranian care contexts could enhance intervention strategies, while comparative trials of ivermectin-permethrin combinations versus alternative treatments would strengthen clinical guidelines (Mitchell et al., 2024). The implementation of digital tools for real-time detection in low-resource settings would further support global efforts (Agency, 2025).

In conclusion, this study presented a managed scabies outbreak in an Iranian nursing home, affecting four female residents (one case with CS and three cases with OS) and resolved through timely diagnosis, combined therapy, prophylaxis, and environmental strategies, with no involvement from staff or recurrences over one year. These findings underscore the effectiveness of integrated approaches in vulnerable settings, addressing a disease that resulted in 5.3 million Disability-Adjusted Life Years (DALYs) globally in 2021 (Li et al., 2024).

## CRediT authorship contribution statement

**Hamed Behniafar:** Writing – original draft, Methodology. **Kiana Yahyaei:** Methodology. **Arian Taghdiri:** Methodology. **Hamideh Mohammadzadeh:** Writing – review & editing, Conceptualization. **Hossein Pazoki:** Writing – review & editing, Project administration, Conceptualization.

## Ethical approval

Ethical approval for this study was received from the Ethics Committee of Gonabad University of Medical Sciences (IR.GMU.REC.1403.116). Before sample collection, written informed consent was obtained from the participants. All procedures were conducted in accordance with the guidelines established by the committee.

## Funding

No funds, grants, or other support were received for conducting this study.

## Declaration of competing interest

The authors declare no competing interests.

## Data Availability

All data for this article are available from the corresponding author and can be provided if needed.

## References

[bb0005] Absil G. (2022). Scabies and therapeutic resistance: current knowledge and future perspectives. JEADV Clin. Pract..

[bb0010] Agency (2025). UK Health Security Agency (UKHSA) Guidelines for the Management of Scabies Cases and Outbreaks in Long-term Care Facilities and Other Closed Settings. https://www.gov.uk/government/publications/scabies-management-advice-for-health-professionals/ukhsa-guidance-on-the-management-of-scabies-cases-and-outbreaks-in-long-term-care-facilities-and-other-closed-settings.

[bb0015] Agyei M. (2024). An unusual case of crusted scabies in an immunocompetent adult: a case report. Clin. Case Reports.

[bb0020] Al-Soufi L. (2025). Crusted scabies in a malnourished patient: a rare case report. Clin. Med. Insights.

[bb0025] Andriantsoanirina V. (2014). Molecular survey of knockdown resistance to pyrethroids in human scabies mites. Clin. Microbiol. Infect..

[bb0030] Bergamin G. (2024). A systematic review of immunosuppressive risk factors and comorbidities associated with the development of crusted scabies. Int. J. Infect. Dis..

[bb0035] Braun M. (2020). The challenge of diagnosing scabies in the elderly: a case and a novel therapeutic approach. Int. J. Women's Dermatol..

[bb0040] Cassell J.A. (2018). Scabies outbreaks in ten care homes for elderly people: a prospective study of clinical features, epidemiology, and treatment outcomes. Lancet Infect. Dis..

[bb0045] Davis J.S. (2013). A novel clinical grading scale to guide the management of crusted scabies. PLoS Negl. Trop. Dis..

[bb0050] Debash H. (2024). Parasitological prevalence of scabies and secondary bacterial infections among scabies suspected patients at Borumeda General Hospital, Northeast Ethiopia. BMC Infect. Dis..

[bb0055] Derek Richard S. (2002). Prevalence of skin disease among nursing home patients in southern Taiwan. Int. J. Dermatol..

[bb0060] El-Moamly A.A. (2021). Scabies as a part of the World Health Organization roadmap for neglected tropical diseases 2021–2030: what we know and what we need to do for global control. Trop. Med. Health.

[bb0065] Engelman D. (2020). The 2020 international alliance for the control of scabies consensus criteria for the diagnosis of scabies. Br. J. Dermatol..

[bb0070] Engelman D. (2021). A framework for scabies control. PLoS Negl. Trop. Dis..

[bb0075] Giorgio C.M. (2025). Post-COVID-19 resurgence of scabies in Campania, Italy: the hidden burden and challenges in surveillance. Dermatol. Rep..

[bb0080] Gupta S. (2024). Prevalence and determinants of scabies: a global systematic review and meta‐analysis. Trop. Med. Int. Health.

[bb0085] Hewitt K., Nalabanda A., Cassell J. (2015). Scabies outbreaks in residential care homes: factors associated with late recognition, burden and impact. A mixed methods study in England. Epidemiol. Infect..

[bb0090] Jung J. (2025). Management of a large nosocomial outbreak from an index of crusted scabies in a tertiary care hospital, 2023: a retrospective observational study. J. Korean Med. Sci..

[bb0095] Khoobdel M. (2022). Scabies as a neglected tropical disease in Iran: a systematic review with meta-analysis, during 2000–2022. J. Arthropod. Borne Dis..

[bb0100] Lee M.H. (2020). A systematic review on the causes of the transmission and control measures of outbreaks in long-term care facilities: back to basics of infection control. PLoS One.

[bb0105] Li J., Liu Z., Xia X. (2024). The disability-adjusted life years (DALYs), prevalence and incidence of scabies, 1990–2021: a systematic analysis from the Global Burden of Disease Study 2021. PLoS Negl. Trop. Dis..

[bb0115] Liu J.M. (2016). The effects of climate factors on scabies. A 14-year population-based study in Taiwan. Parasite.

[bb0120] Matthews A. (2021). Prevalence of scabies and impetigo in school-age children in Timor-Leste. Parasit. Vectors.

[bb0125] Mayer K., Biedermann T., Posch C. (2022). European scabies challenge: what about permethrin‐resistant mites?. J. Eur. Acad. Dermatol. Venereol..

[bb0130] Mazumder A., Mehrmal S., Chaudhry S.B. (2022). Dermatologic needs of Afghan refugees. JAAD Int..

[bb0135] Meletis G. (2018). Is the simple saline mount technique more effective than potassium hydroxide for the microscopic detection of Sarcoptes scabiei?. J. Parasitol..

[bb0140] Mitchell E. (2024). Scabies: current knowledge and future directions. Front. Trop. Dis..

[bb0145] Morrison E. (2019). Do we know how scabies outbreaks in residential and nursing care homes for the elderly should be managed? A systematic review of interventions using a novel approach to assess evidence quality. Epidemiol. Infect..

[bb0150] Niode N.J. (2022). Crusted scabies, a neglected tropical disease: case series and literature review. Infect. Dis. Rep..

[bb0155] Organization, W.H (2023). Scabie. https://www.who.int/news-room/fact-sheets/detail/scabies.

[bb0160] Park S.W. (2019). Diagnostic value of positive findings of toxoplasma gondii-specific immunoglobulin M serum antibody in uveitis patients to confirm ocular toxoplasmosis. Ocul. Immunol. Inflamm..

[bb0165] Raffi J., Suresh R., Butler D.C. (2019). Review of scabies in the elderly. Dermatol. Ther..

[bb0170] Rasti S. (2017). Frequency and Clinical Manifestations of Scabies in Suspected Patients Referred to Health Centers of Kashan, Central Iran (2010–2014). Zahedan J. Res. Med. Sci..

[bb0175] Sanei-Dehkordi A. (2021). Risk factors associated with scabies infestation among primary schoolchildren in a low socio-economic area in southeast of Iran. BMC Pediatr..

[bb0180] Saraj W.A. (2023). Scabies outbreak in Afghanistan calls for urgent international response. Razi Int. Med. J..

[bb0185] Shoukat Q. (2023). Sight the mite: a meta-analysis on the diagnosis of scabies. Cureus.

[bb0190] Smith D.R. (2002). Prevalence of skin disease among nursing home staff in southern Taiwan. Ind. Health.

[bb0195] Spaziante M. (2025). Post-COVID-19 resurgence of scabies’ cases in the Lazio Region, Italy: a new emerging public health threat?. Infect. Dis. Poverty.

[bb0200] Sunderkötter C. (2016). S1 guidelines on the diagnosis and treatment of scabies–short version. J. Deutschen Dermatol. Gesellschaft.

[bb0205] Takeda A., Ishibashi T., Sonoda K.-H. (2017). Epidemiology of uveitis, caused by HTLV-1, toxoplasmosis, and tuberculosis; the three leading causes of endemic infectious uveitis in Japan. Ocul. Immunol. Inflamm..

[bb0210] Tavoletti G. (2025). Scabies: an updated review from epidemiology to current controversies and future perspectives. Travel Med. Infect. Dis..

[bb0215] Thomas J. (2017). Scabies-An ancient itch that is still rampant today. J. Clin. Pharm. Ther..

[bb0220] Thomas C. (2020). Ectoparasites: scabies. J. Am. Acad. Dermatol..

[bb0225] Tsoi S.K. (2021). Estimation of scabies prevalence using simplified criteria and mapping procedures in three Pacific and southeast Asian countries. BMC Public Health.

[bb0230] Uzun S. (2024). Clinical practice guidelines for the diagnosis and treatment of scabies. Int. J. Dermatol..

[bb0235] Veraldi S. (2023). Pseudoresistance to permethrin in scabies. J. Infect. Dev. Count..

[bb0240] Walker S.L. (2020). A community-based validation of the International Alliance for the Control of Scabies Consensus Criteria by expert and non-expert examiners in Liberia. PLoS Negl. Trop. Dis..

[bb0245] Walton S.F., Currie B.J. (2007). Problems in diagnosing scabies, a global disease in human and animal populations. Clin. Microbiol. Rev..

[bb0250] White L. (2016). The management of scabies outbreaks in residential care facilities for the elderly in England: a review of current health protection guidelines. Epidemiol. Infect..

[bb0255] Yamaguchi Y. (2024). Investigating the epidemiology and outbreaks of scabies in Japanese households, residential care facilities, and hospitals using claims data: the Longevity Improvement & Fair Evidence (LIFE) study. IJID Reg..

